# Prophylactic and Ameliorative Effects of PPAR-γ Agonist Pioglitazone in Improving Oxidative Stress, Germ Cell Apoptosis and Inflammation in Gentamycin-Induced Testicular Damage in Adult Male Albino Rats

**DOI:** 10.3390/antiox11020191

**Published:** 2022-01-19

**Authors:** Karima El-Sayed, Dina A. Ali, Shymaa Ahmed Maher, Dalia Ghareeb, Samy Selim, Sarah Albogami, Eman Fayad, Eman Kolieb

**Affiliations:** 1 Physiology Department, Faculty of Medicine, Suez Canal University, Ismailia 41522, Egypt; karima_ahmed@med.suez.edu.eg; 2 Clinical Pharmacology Department, Faculty of Medicine, Suez Canal University, Ismailia 41522, Egypt; dina_abdel-karim@med.suez.edu.eg; 3 Medical Biochemistry and Molecular Biology Department, Faculty of Medicine, Suez Canal University, Ismailia 41522, Egypt; shimaa.maher@med.suez.edu.eg; 4 Center of Excellence in Molecular and Cellular Medicine (CEMCM), Faculty of Medicine, Suez Canal University, Ismailia 41522, Egypt; 5 Clinical Pathology Department, Faculty of Medicine, Suez University, Suez 41522, Egypt; dalia.ghareeb@med.suezuni.edu.eg; 6 Department of Clinical Laboratory Sciences, College of Applied Medical Sciences, Jouf University, Sakaka 72341, Saudi Arabia; sabdulsalam@ju.edu.sa; 7 Department of Biotechnology, Faculty of Sciences, Taif University, Taif 21944, Saudi Arabia; dr.sarah@tu.edu.sa (S.A.); e.esmail@tu.edu.sa (E.F.)

**Keywords:** testis, gentamycin, PPAR-γ, testosterone, oxidative stress, inflammatory cytokines

## Abstract

Peroxisome proliferator-activated receptor gamma (PPAR-γ) is ubiquitously expressed in testicular tissue and plays a crucial role in regulating various physiological processes. Pioglitazone (PIO) is one of the PPAR-γ agonists, having anti-oxidant and anti-inflammatory effects. Patients on gentamycin treatment may undergo serious side effects such as testicular damage. To the best of our knowledge, this was the first study to investigate the possible protective anti-inflammatory and anti-apoptotic effects of PIO on gentamycin-induced testicular damage. Fifty adult male Wistar albino rats included in the study as the control group (CTL) received normal saline; a gentamycin-induced testicular damage group (GM) received gentamycin (100 mg/kg); PIO5, PIO10, PIO20 groups received PIO at a dose of 5, 10, and 20 mg/ kg, respectively, for 21 days, and gentamycin was started at day 15 of the experiment for 6 days. The parameters of spermatozoa and histopathological alterations in the testes were significantly improved in the PIO20 group. Moreover, MDA levels, inflammatory mediators, and apoptotic Bax expression were decreased. The activity of glutathione peroxidase, catalase, total antioxidant capacity, and anti-apoptotic Bcl-2 genes expression were increased. It was concluded that PIO20 could protect against gentamycin-induced testicular damage in Wistar rats through its anti-oxidant, anti-inflammatory, and antiapoptotic effects.

## 1. Introduction

Infertility is a common health problem with psychological and medical implications. Globally, it is estimated that infertility affects about 12% of couples, with the male factor being a main or contributing cause in around 50% of infertility cases [1]. Several factors have been implicated in the decline of male fertility, including environmental toxicants and xenobiotics which have harmful effects on normal spermatogenesis, normal production of spermatozoa, and semen quality [2].

Gentamycin is an antibiotic in the aminoglycosides group. Although gentamycin is used in the treatment of gram-negative bacterial infections [3], it can produce organ toxicity, including testicular toxicity which limits its therapeutic uses. Gentamycin impairs the motility of spermatozoa and can also cause apoptosis in rat testes, resulting in testicular failure [4].

Oxidative stress plays an important role in the pathogenesis of several system disorders including reproductive disorders; it can result in the defective production of spermatozoa and infertility. Oxidative stress has been reported in several studies on testicular toxicity induced by gentamycin, and several antioxidants have been used to protect against gentamycin-induced testicular damage [5,6].

Gentamycin is also known to reduce the count, viability, and motility of spermatozoa by decreasing the antioxidant enzyme levels resulting in increasing free radical formation and lipid peroxidation [7]. Free radicals induce oxidative damage to spermatozoa which play a critical role in increasing poor function of the spermatozoa and infertility. Spermatozoa are susceptible to oxidative stress-induced damage due to the presence of large polyunsaturated fat content in their membranes [8].

PPAR-γ is a ligand-regulated nuclear receptor (PPAR). These receptors form heterodimers with the retinoid X receptors (RXRs) to produce functional transcription factors that are involved in the trans-activation of sequential key genes during energy homeostasis and cellular differentiation. Moreover, PPAR and RXR transcripts encoding members of the PPAR and RXR nuclear receptor family reach maximum levels of expression in the germ cells during the early meiotic stages of spermatogenesis [9].

PPAR-γ has an important role in the regulation of energy homeostasis. It modulates the hypothalamic-pituitary-gonadal (HPG) axis, and at the same time, it is commonly regulated by HPG. In humans, PPAR-γ protein is expressed in ejaculated spermatozoa, germ cells, and Sertoli cells [9]. The peak of PPAR-γ levels occurs at a late stage of spermatogenesis, concomitant with increased levels of RXRβ and RXRγ expression. PPAR-γ/RXRγ heterodimeric transcription factor complexes, which are expressed in mature Sertoli cells, up-regulate lipid metabolic target genes in these cells, providing them with enough energy to support spermatogenesis. In addition, male fertility can be blocked by the inactivation of genes involved in lipid metabolism [10].

Pioglitazone is a potent and selective agonist for the PPAR-γ receptor which is part of the steroid and thyroid superfamily of nuclear receptors [11]. The mechanism of action of PIO involves its binding to the PPAR-γ nuclear receptor which acts as a transcription factor upon activation. It regulates the transcription and expression of specific genes by two mechanisms: trans-activation, a DNA-dependent mechanism, and trans-repression, DNA-independent mechanisms. In trans-activation, PIO binds and activates PPAR-γ, then forms a heterodimer with the RXR, and they bind to specific peroxisome proliferators response elements (PPRE) on several key target genes involved in the carbohydrate and lipid metabolism in many tissues, including muscle, adipose tissue, and liver which increases the circulating lipid and glucose levels. Saturated fatty acids have pro-inflammatory effects in several cell types, so PIO has a potential effect on the circulating levels of saturated fatty acids that could indirectly affect inflammation [12].

The second mechanism, trans-repression, involves interfering with other transcription-factor pathways in a DNA-independent manner, such as the stoppage of other transcription factors such as Nuclear Factor Kappa B (NF-κB). This partly explains the anti-inflammatory actions of PPAR-γ agonists [13,14]. After binding PIO to PPAR-γ and its activation, there is enhancement of the differentiation and the proliferation of pre-adipocytes into adipocytes that are capable of lipid uptake, expression of hormones, and cytokines, particularly in peripheral and subcutaneous fat [15]. The increased uptake of fatty acids in peripheral and subcutaneous adipocytes results in a “lipid steals” phenomenon. This would lead to a reduction of the circulating fatty acids and triglycerides concentration in muscle and liver, increased sensitivity of insulin through enhancement of glucose utilization in muscles, reduction of hepatic glucose output, and altering “adipokines” such as adiponectin, resistin, and TNF-α. The reduction of TNF-α, a pro-inflammatory cytokine, participates in the anti-inflammatory action [16].

Inflammation is characterized by the activation of macrophages and monocytes at the injury site, which increases pro-inflammatory mediators release such as TNF-α, IL-6, and IL-1β which stimulate the production of cyclooxygenase (COX) products [17]. Pioglitazone has an anti-inflammatory action as it plays an important role in the immune response. Moreover, PIO has the ability to inhibit the expression of inflammatory cytokines and to direct the differentiation of immune cells toward anti-inflammatory phenotypes [18].

To the best of our knowledge, there are no data describing the testicular-protective potential role of PIO against gentamycin-induced testicular damage in rats. Therefore, the aim of the study was to investigate the testicular-protective effects of PIO at 5, 10, and 20 mg/kg against gentamycin-induced testicular damage and the antioxidant activity of PIO as well as underlying anti-inflammatory and anti-apoptotic mechanisms in male Wistar albino rats. Study hypothesis: PIO at 5, 10, and 20 mg/kg has testicular protective effects by decreasing testicular histopathological changes and modulating the parameters of spermatozoa, testicular oxidative stress, inflammatory, and apoptotic markers in gentamycin-induced testicular damage in male Wistar albino rats.

## 2. Materials and Methods

### 2.1. Experimental Animals

In the present study, fifty adult male Wistar albino rats (200–250 g) were obtained from Faculty of Veterinary Medicine, Suez Canal University, Egypt. The rats were maintained under controlled room temperature of 22 ± 3 °C with 12 h light/dark cycles and the humidity level of 50–60%. A standard pellet chow and fresh tap water were available and libitum. Animals were left to adjust for two weeks before the study starts.

### 2.2. Study Design

As shown in (Table 1); rats were randomly divided into five groups (ten rats/group).

Group I: Control group (CTL) in which rats received no treatment, only normal saline 10 mL/kg/day by oral gavage for 21 days (0.3 mL/rat).

Group II: Gentamycin induced testicular damage group (GM) in which rats received gentamycin (GM, Memphis Pharma Production, Egypt) by intra-peritoneal injection (I.P), at a dose of 100 mg/kg/day, for 6 days. The dose of GM was matched with previous studies that confirmed multi-organ systems damage in rats [3].

Group III: PIO 5 group in which rats received PIO treatment at a dose of (5 mg/kg/day) as a single daily oral dose by gastric gavage for 21 days, with GM started from day 15 of PIO treatment and continued for 6 more days [19].

Group IV: PIO 10 group, this group received PIO treatment at a dose of (10 mg/kg/day) as a single daily oral dose by gastric gavage for 21 days, with GM started from day 15 of PIO treatment and continued for 6 days [19].

Group V: PIO 20 group, this group received PIO treatment with a dose of (20 mg/kg/day) once/ day oral dose by gastric gavage for 21 days, with GM started from day 15 of PIO treatment and continued for 6 days [19].

Pioglitazone was selected based on the results of previous studies which demonstrated that it has an anti-inflammatory and antioxidant effects. It prevents and protects against renal dysfunction in gentamycin induced nephrotoxicity in rats [20]. Moreover, PIO only administration group showed quite similar results to the control group regarding oxidant level and antioxidant activities in gentamycin induced nephrotoxicity in rats [21], therefore, PIO-only administration group wasn’t added to the above-mentioned study groups.

Pioglitazone hydrochloride powder was provided by Medical Union Pharmaceutical Co. (Abu-Sultan, Egypt). It was prepared and suspended in 0.2% carboxymethyl cellulose (CMC) aqueous solution in 3 different concentrations (0.25, 0.5, and 1%) representing PIO’s different doses 5, 10, and 20 mg/kg, respectively. The amount of one-shot is about 0.4 mL for a rat weighing 200 g.

Pioglitazone was dissolved in methylcellulose [22], It was stated that PIO showed good absorption from the gastrointestinal tract, about 96% in rats. Four hours after administration, the plasma concentration of PIO reached its peak with a half-life of 2.6 h [23].

### 2.3. Collection of Samples

The experimental animals of each group were weighed the day following the end of treatment. The rats were anesthetized with ketamine (50 mg/kg) and xylazine (5 mg/kg). The blood samples were taken through cardiac puncture using a 5-gauge syringe, and then they were centrifuged at 3000× *g* for 10 min. Serum was separated and stored at −80 °C till analysis. After that, rats were sacrificed by exsanguination (aortic dissection), immediately testes were removed weighted and cauda epididymis was dissected from each testis. One testis (right testis) was fixed in Bouin’s for histopathological analysis. The other testis (left testis) was washed by phosphate buffer saline (PBS) then was weighed, cut into two parts. The first part (30 mg) was kept at −80 °C to be used for RNA extraction, for analysis of Bax and BCL-2 gene expression as an indication of apoptosis. The second part was homogenized in PBS (pH = 7–7.2) using a Teflon homogenizer (Glas-Col; Vernon Hills, IL, USA). The homogenates were centrifuged at 20,000× *g* for 15 min. Next, they were stored at −80 °C to be used for measuring the levels of oxidative stress markers and antioxidant enzyme activity in testicular tissue.

### 2.4. Assessment of Testis Weight and Sperm Parameters (Count and Motility)

Analysis of the spermatozoa was managed according to Catanzariti et al., 2010 [24]. Samples of the spermatozoa were taken from the cauda epididymis. They were placed in a petri dish, minced and then incubated in 15 mL NA citrate as a media for 30 min at 37 °C in 5% CO_2_ incubator to allow the sperm to swim in the media.

For detection of sperm count, homogenization of the right cauda epididymis was performed in a glass-Teflon homogenizer with 10 mL saline for 2 min at 3000× *g*. After that, the suspension of spermatozoa was sited into a hemocytometer (Neubauer, Seligenstadt, Germany). A light microscope was used to manually count the remained sperm heads that were not homogenized (Leica, Wetzlar, Germany).

At first, the number of spermatozoa from both chambers of the haemocytometer was averaged. Then, the mean of counted spermatozoa divided by volume within which they were counted (volume of 25 large square = 100 nL). The obtained sperm concentration was the number of spermatozoa per nL, which equals millions of sperms/mL (sperm/10^−9^ L = sperm × 106/10^−3^ L).

To obtain the number of spermatozoa per cauda epididymis, sperm concentration was multiplied by the number of BWW medium volume used in swim up.

An example of spermatozoa number calculation: 15 mL solution of BWW media was added to epididymal spermatozoa sample (swim-up method). Counting on one chamber produced 320, whereas in the other counting chamber is obtained 280. Both these results are summed and divided by two to get the average, obtained number 300.

To obtain the concentration of spermatozoa per nL, the average spermatozoa number of both chambers was divided by 100, to obtain the numbers 3 × 106 sperm per mL of sperm suspension. To obtain the number of spermatozoa per cauda epididymis, spermatozoa concentration (3 × 106 sperm per mL) was multiplied by the volume of BWW used to swim up (15 mL) yields: 30 × 106 = 3180 spermatozoa per cauda epididymis of rat [25].

For assessment of motility, seminal fluid was obtained from the left cauda epididymis by cutting with a pair of scissors and then immediately placed in a Petri dish having 5 mL Hanks’ balanced salt solution (pH 7.2) with 5 mg/mL of bovine serum albumin and incubated for 5 min at 37 °C. A microscope with a stage warmer was used to observe motility. Spermatozoa were considered motile if they showed any movement at all. Smears of sperm suspensions were stained with 1% Eosin Y and allowed to air dry on a glass slide [26].

### 2.5. Hormonal Assay

Serum levels of testosterone, follicle-stimulating hormone (FSH) and luteinizing hormone (LH) were measured using specific ELISA kits, Rat Testosterone ELISA Kit; Biosource Europe S.A, Belgium (Catalog Number MBS282195), Rat FSH ELISA Kit Cusabio (China) (Cat. No. CSB-E06869r), Rat LH ELISA Kit (Cat. No. CSB-E12654r) according to manufacturer’s instructions.

### 2.6. Measurement of Serum Inflammatory Markers

Serum IL-6, and TNF-α levels were assessed using enzyme-linked immunosorbent assay (ELISA) kits (R & D Systems, Minneapolis, MN, USA) according to the manufacturer’s instructions. Rat IL-6 Immunoassay (Cat. No. R6000B) & Rat TNF-α Immunoassay (Cat. No. RTA00).

### 2.7. Measurement of Oxidative Stress & Antioxidant Markers

Total antioxidant capacities (TAC) together with the level of peroxide, that reflect total oxidative stress status (TOS), were measured in serum using a colorimetric assay by (Biodiagnostics, Tahreer St, Dokki, Giza, Egypt) with TAC kit (Cat. No. TA 25) Peroxide kit (Cat. No. HP25).

Levels of oxidative stress markers; malondialdehyde (MDA) (marker for lipid per-oxidation) and activities of antioxidant enzymes catalase, glutathione peroxidase in testicular tissue homogenate were measured according to the manufacturer’s instructions using a colorimetric assay by (Biodiagnostics, Tahreer St, Dokki, Giza, Egypt) with the following kits: MDA kit (Cat. No. MD 2529), catalase (Cat. No. CA 25 17), glutathione peroxidase (Cat. No. GP 2524).

### 2.8. RNA Extraction and Quantitative RT-PCR Detection of Testicular BAX & BCL2 Genes

The RNA was extracted from testicular tissue using RNeasy Mini Kit (cat no 74104, Qiagen, Germany). RNA was measured using Nano drop 8000 (Thermo Scientific, Waltham, MA, USA) and reverse transcription was carried out using (Quanti Tect Reverse Transcription Kit (Cat. No. 205311, Qiagen, Hilden, Germany), mRNA expression level of anti-apoptotic and apoptotic markers; Bcl-2 and Bax, respectively, was detected by SYBR Green PCR master mix (QuantiTect SYBR Green PCR Kit (Cat. No. 204141, Qiagen, Hilden, Germany) and the used primers for PCR reaction as mentioned in (Table 2). The 2^-ΔΔCt^ method of analysis was used to analyze the results according to [27].

### 2.9. Histopathological Examination

The right testis was fixed in Bouin’s solution, for 24 h, processed in ascending alcohol concentrations and embedded in paraffin. After that, tissues were fixed in paraffin and sectioned at 4 mL thickness, deparaffinized, and rehydrated using standard techniques. The SNT sections were stained with hematoxylin-eosin stain for microscopic examination. All sections were examined blindly with a light microscope by a pathologist according to Johnsen’s scoring system of testicular tissue (Table 3) [28].

If the testes were fixed in 10% neutral buffered formalin, the formalin would cause severe shrinkage of the Sertoli and germ cells within the tubules, which seriously compromise the pathologist’s ability to detect any changes in them. Fixation of testes with Bouin’s overcome the shrinkage problem and maintained the architectural integrity of the SNT. It also prevented the disruption of the delicate germ cell sertoli cell junction. Bouin’s contained formalin but also it contained citric acid and acetic acid which led to swelling of the cells, helping to counteract the shrinkage induced by formalin. Bouin’s provided an excellent cellular and nuclear morphology of germ and Sertoli cells [29,30].

A score from 1 to 10 was given, according to the presence or absence of germ cells [31]. SNT diameter was measured at 200× magnifications; besides, the epithelium thickness was assessed at 400× magnification using the light microscope. After selection, rounded tubules were cut transversely and the average of tubule diameters was calculated depending on two spans of each tubule, one vertical to the other [16]. For the measurements in oblique sections, the minor axis was taken [32,33].

Images were analyzed using Image J software (version 1.50b, USA) [34]. Thirty tubules for each animal were graded using a 400× magnification and each tubule was given a score from 1 to 10 based on the presence or absence of germ cell types in the testicular seminiferous tubules such as spermatozoa, spermatids, spermatocyte, spermatogonia, germ cells and Sertoli cells to evaluate histology. A higher Johnsen’s score indicates a better status of spermatogenesis, while a lower score refers to more severe dysfunction. Score 1 means no epithelial maturation is considered for the tubules with complete inactivity while a score 10 means full epithelial maturation is considered for the tubules with maximum activity [31].

### 2.10. Statistical Analysis

One-way analysis of variance (ANOVA) was used for comparisons among groups then it was followed by Tukey’s post-hoc test. Data were represented as means + standard errors of mean. *p* values < 0.05 showed statistical significance. Data were analyzed using Origin Pro software (version 8.0724, Origin Lab Corporation, Northampton, MA, USA). N refers to the number of rats.

### 2.11. Ethical Statement

The study was conducted according to the guidelines of the Declaration of Helsinki and approved by the Institutional Re Ethics Committee of Faculty of Medicine, Suez Canal University, Ismailia, Egypt, approval number Research 4506 #.

## 3. Results

### 3.1. Body Weight, Testis Weight and Relative Testis Weight

The body weight of animals did not show any significant changes between different groups. Induction of testicular damage in male rats with gentamycin (GII, GM) caused a significant reduction (*p* < 0.01) in absolute testis weight compared to control animals (GI, CTL). Treatment with pioglitazone (PIO 5 GIII, PIO 10 GIV, and PIO 20 GV) caused a significant increase in absolute testis weight compared to the gentamycin treated group (GII, GM) (*p* < 0.05, *p* < 0.01, *p* < 0.01). Absolute testis weight showed a non-significant difference between the control group (GI, CTL) and GV, PIO 20, with (*p* > 0.05). However, there was a significant difference between GIII, PIO 5 and GV, PIO 20 regarding testicular weight, with (*p* < 0.05). 

Relative testis weight was significantly decreased (*p* < 0.05) in gentamycin treated group GM compared to the control group CTL, while with treated group GIV (PIO10) and GV (PIO 20) there was a significant increase in relative testis weight (*p* < 0.05 & *p* < 0.01) respectively, GIII (PIO 5) showed a non- significant difference compared to GII (GM) (*p* > 0.05).

Relative testis weight showed insignificant difference between the control group (GI, CTL) and GV, PIO 20, with (*p* > 0.05) and between GIII, PIO 5 and GV, PIO 20, with (*p* > 0.05), as shown in (Table 4).

### 3.2. Sperm Parameters

Treatment of male rats with gentamycin (GII, GM) caused a significant reduction (*p* < 0.01) in sperm count, sperm motility, and sperm viability compared to control animals (GI, CTL). Treated groups with pioglitazone in different doses (GIII PIO 5, GIV PIO 10, GV PIO 20) showed significant improvement in sperm parameters compared (GII, GM) (Table 5).

### 3.3. Pituitary-Testicular Hormonal Axis

**Figure 1:**
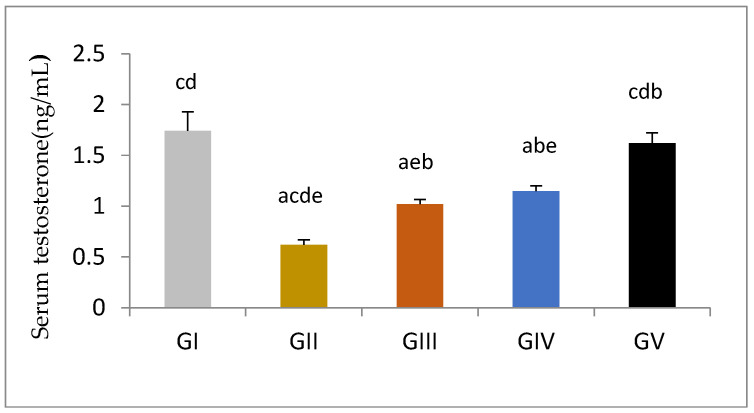
Serum testosterone level measured by ELISA in different groups, GI (CTL): normal control, GII (GM): gentamycin induced testicular damage, GIII (PIO5): pioglitazone treated (5 mg/kg/day), GIV (PIO10): pioglitazone treated (10 mg/kg/day) and GV (PIO20): pioglitazone treated (20 mg/kg/day). Values are as mean ± SEM. (a) compared to control group, (b) compared to a diseased group, (c) compared to PIO 5 (GIII) group, (d) compared to PIO 10 (G IV) group, (e) compared to PIO 20 (GV) group. Serum LH and FSH were significantly elevated in response to gentamycin treatment in (GII, GM) compared to (GI, CTL) (*p* < 0.01). While the treated groups (GIII, GIV) showed a significant reduction in serum LH and FSH compared to GII, GM (*p* < 0.01) for both. GIV, PIO10 and GV, PIO 20 showed a non- significant difference in LH level compared to GI, CTL (*p* ≥ 0.05, *p* > 0.05) respectively, while only GV, PIO20 showed a non- significant difference in FSH level compared to GI, CTL (*p* > 0.05) (Table 6).

Serum testosterone was significantly reduced in response to gentamycin induction in (GII, GM) compared to GI, CTL (*p* < 0.01). While the treated groups (GIII, GIV) showed a significant increase in serum testosterone compared to GII, GM (*p* < 0.01), group (GV, PIO20) showed a non -significant difference compared to (GI, CTL) (*p* > 0.05) (Figure 1).

Serum LH and FSH levels showed non-significant differences between the control group (GI, CTL) and GV, PIO 20, with (*p* > 0.05) for both. However, there was a significant difference between GIII, PIO 5 and GV, PIO 20, with (*p* < 0.01) for both.

### 3.4. Inflammatory Markers

On studying the effects of gentamycin and different doses of pioglitazone on inflammatory markers, the study found that the level of inflammatory markers TNF-α and IL-6 in the gentamycin induced testicular damage group (GII, GM) was significantly increased compared to (GI, CTL), while the level of these markers in pioglitazone treated groups show significant reduction compared to (GII, GM) (*p* ˂ 0.01) (Table 7).

TNF-α and IL-6 showed a significant difference between control group (GI, CTL) and GV, PIO 20, with (*p* < 0.01). Similarly, there was a significant difference between GIII, PIO 5 and GV, PIO 20, with (*p* < 0.01).

### 3.5. Oxidative & Antioxidant State

#### 3.5.1. Oxidative Stress (Total Antioxidant Capacity (TAC) and Total Oxidant Status (TOS) Serum Levels

Induction of testicular damage in male rats with gentamycin (GII, GM) caused a significant decrease in TAC (*p* < 0.01) compared to the control group (GI, CTL), with a significant increase in TOS (*p* < 0.01). Groups GIV (PIO10) & GV (PIO20) showed a significant increase in TAC level compared to GII (GM) (*p* < 0.01), while GIII(PIO5) showed a non- significant increase in TAC level compared to GII (*p* = 0.44), GIII, GIV and GV showed a significant decrease in TOS and OSI compared to GII (GM) (*p* < 0.01) (Table 8).

TAC showed a non-significant difference between the control group (GI, CTL) and GV, PIO 20, with (*p* > 0.05). However, there was a significant difference between GIII, PIO 5 and GV, PIO 20, with (*p* > 0.05). TOS showed a non-significant difference between the control group (GI, CTL) and GV, PIO 20, with (*p* > 0.05). However, there was a significant difference between GIII, PIO 5 and GV, PIO 20, with (*p*< 0.01).

#### 3.5.2. Testicular Tissue Lipid Peroxide Production

**Figure 2:**
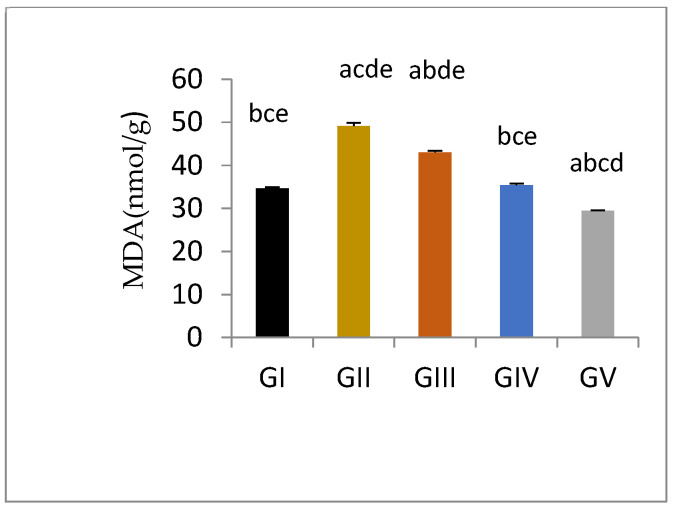
Tissue MDA level measured spectrophotometrically in different groups, GI, CTL: normal control, GII, GM: gentamycin induced testicular damage, GIII, PIO5: pioglitazone treated (5 mg/kg/day), GIV, PIO10: pioglitazone treated (10 mg/kg/day) and GV, PIO20: pioglitazone treated (20 mg/kg/day). Values are expressed as mean ± SED, (^a^) compared to control group, (^b^) compared to a diseased group, (^c^) compared to PIO 5 (GIII) group, (^d^) compared to PIO 10 (G IV) group, (^e^) compared to PIO 20 (GV) group.

Malondialdehyde is the product of polyunsaturated fatty acid and is generally used as a lipid per-oxidation marker. Induction of testicular damage in male rats in gentamycin treated groups (GII, GM) caused a significant increase in MDA level (*p* < 0.01) compared to control group (GI, CTL). While groups treated with pioglitazone showed a significant reduction in tissue MDA level compared to GII (GM) (*p* < 0.01), with increasing PIO dose; the level of MDA was decreased to approach normal. Group GIV (PIO10) showed an in-significant difference compared to GI (CTL)(*p* > 0.05), while GV (PIO20) showed a significant reduction in MDA level compared to GI (CTL) (*p* < 0.05) (Figure 2, Table 9).

#### 3.5.3. Testicular Antioxidant Activities

Induction of testicular damage in male rats with gentamycin(GII, GM) caused a significant reduction in tissue catalase, glutathione peroxidase activities compared to control group (GI, CTL) (*p* < 0.01). While treated groups with pioglitazone at a dose of 5 mg (GIII) showed a non-significant increase in tissue catalase and glutathione peroxidase activities compared to gentamycin treated group (GII) (*p* > 0.05), with an increasing dose of pioglitazone in GIV(PIO10) and GV(PIO20), there were a significant increase in tissue catalase and glutathione peroxidase activities compared to gentamycin treated group (GII, GM) (*p* < 0.01).

**Figure 3:**
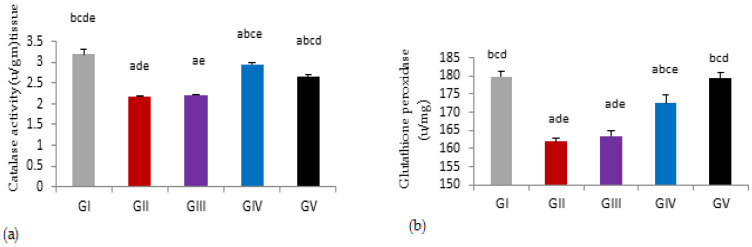
(**a**) Tissue activities of glutathione peroxidase measured spectrophotometrically in different groups. (**b**) Tissue activities of catalase measured spectrophotometrically in different groups, GI, CTL: normal control, GII, GM: gentamycin induced testicular damage, GIII, PIO5: pioglitazone treated (5 mg/kg/day), GIV, PIO10: pioglitazone treated (10 mg/kg/day) and GV, PIO20: pioglitazone treated (20 mg/kg/day). Values are as mean ± SEM, (^a^) compared to control group, (^b^) compared to a diseased group, (^c^) compared to PIO 5 (GIII) group, (^d^) compared to PIO 10 (G IV) group, (^e^) compared to PIO 20 (GV) group.

Tissue catalase activities showed a significant difference between the control group (GI, CTL) and GV, PIO 20, with (*p* < 0.01). Similarly, there was a significant difference between GIII, PIO 5 and GV, PIO 20, with (*p* < 0.01). While glutathione peroxidase activities showed the in-significant difference between the control group (GI, CTL) and GV, PIO 20, with (*p* > 0.05), but showed a significant difference between GIII, PIO 5 and GV, PIO 20, with (*p* < 0.01). (Figure 3, Table 10).

### 3.6. Apoptotic and Anti-Apoptotic Tissue Expression Levels

The expression of the apoptotic gene BAX was significantly increased in gentamycin treated group (GII, GM) (*p* < 0.01) compared to control group (GI, CTL) While groups treated with pioglitazone showed significant reduction in the expression level of BAX gene compared to GII(GM) (*p* < 0.01), with increasing PIO dose; BAX gene expression was further decreased in a dose dependent manner. 

Bax expression showed significant difference between control group (GI, CTL) and GV, PIO 20, with (*p* < 0.01).Similarly there was significant difference between GIII, PIO 5 and GV, PIO 20, with (*p* < 0.01).

**Figure 4:**
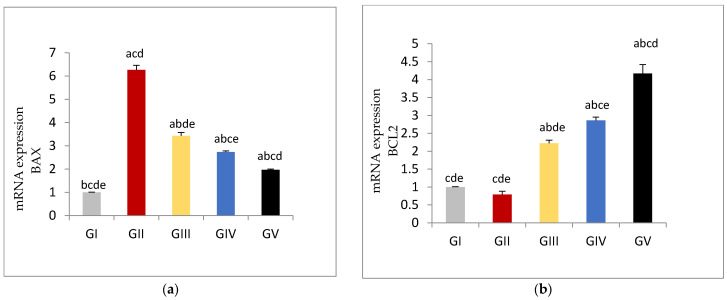
(**a**) mRNA Expression levels of BAX gene in different groups, (**b**) mRNA Expression levels of BCL2 gene in different groups GI, CTL: normal control, GII, GM: gentamycin induced testicular damage, GIII, PIO5: pioglitazone treated (5 mg/kg/day), GIV, PIO10: pioglitazone treated (10 mg/kg/day) and GV, PIO20: pioglitazone treated (20 mg/kg/day). Values are as mean ± SEM (^a^) compared to control group, (^b^) compared to diseased group, (^c^) compared to PIO 5 (GIII) group, (^d^) compared to PIO 10 (G IV) group, (^e^) compared to PIO 20 (GV) group.

On the other hand, in gentamycin treated group (GII, GM) BCL2 expression showed in- significant reduction compared to (GI, CTL). Treated groups with pioglitazone showed a significant rise in the BCL2 expression compared to GII (GM) (*p* < 0.01). BCL2 expression showed a significant difference between control group (GI, CTL) and GV, PIO 20, with (*p* < 0.01). Similarly, there was a significant difference between GIII, PIO 5 and GV, PIO 20, with (*p* < 0.01), (Figure 4, Table 11).

### 3.7. Histopathological Assessment of the Testicular Tissue

#### 3.7.1. Histopathological Assessment

**Figure 5:**
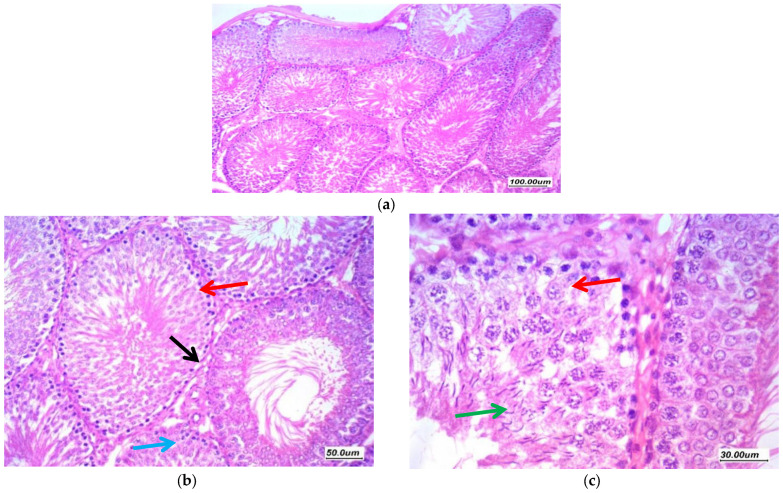
Sections in the testis of a control group (GI, CTL) (**a**) 100×, (**b**) 200×, and (**c**) 400×. Testicular tissue showed regularly arranged seminiferous tubules with thin basement membrane (black arrow) lined by germ cells from basal spermatogonia (red arrow) showing regular maturation to the spermatid and spermatozoa stage. There are scattered Sertoli cells with associated spermatozoa (green arrow). The interstitial tissue showed scattered Leydig cells and thin small vessels (blue arrow).

**Figure 6:**
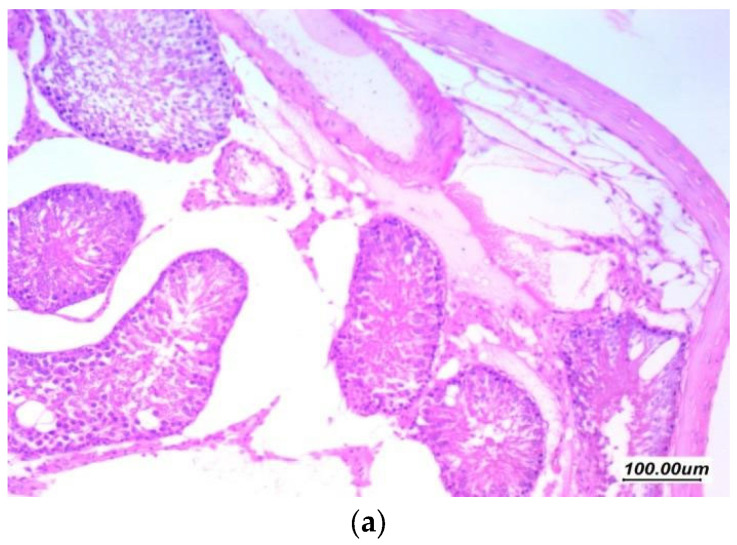

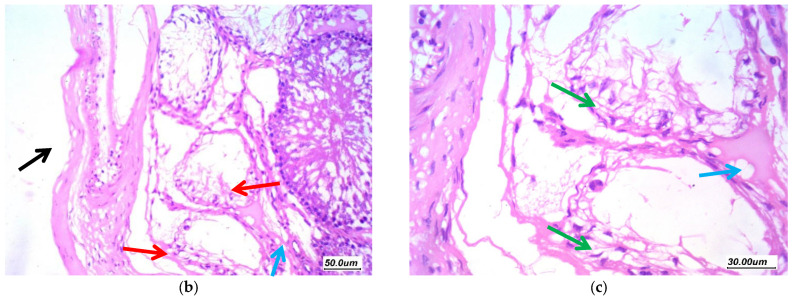
Effect of gentamycin on the histopathological picture of testis specimens in group (GII, GM) (**a**) 100×, (**b**) 200×, and (**c**) 400× Testicular tissue showed markedly distorted seminiferous tubules showing variability in size and shape with irregular contours (red arrow). There is marked distortion of germ cell lining with vacuolar degeneration and shedding (green arrow), loss of maturation of germ cells, with few tubules cystically dilated. There is moderate thickening of basement membrane; there is moderate thickening of tunica (black arrow). The interstitium showed marked edema (blue arrow).

**Figure 7:**
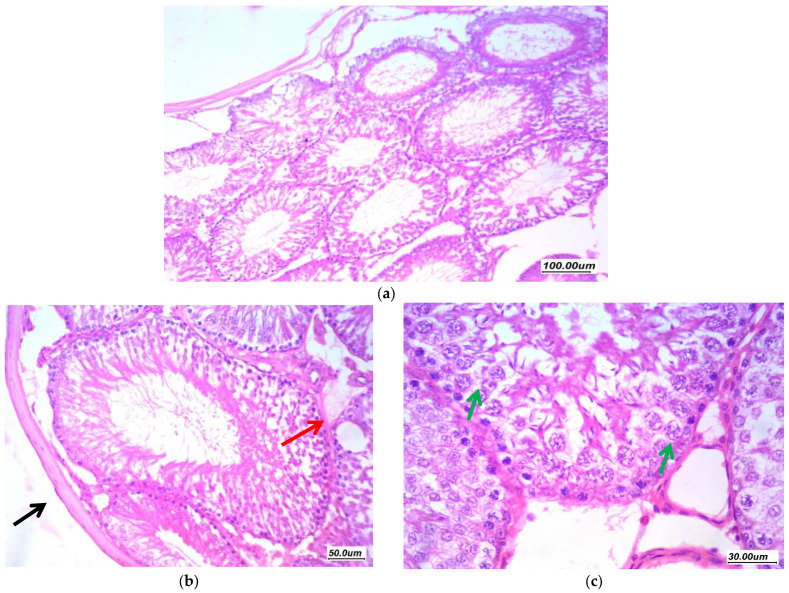
Effect of Pioglitazone (5 mg/kg/day) on gentamycin induced testicular damage in group (GIII, PIO5) (**a**) 100×, (**b**) 200× and (**c**) 400×. There is moderate improvement of changes, tubules showed regular arrangement, moderate residual vacuolar degeneration of germ cells in few tubules (green arrow). Moderate residual edema (red arrow) and focal thickening of tunica (black arrow).

**Figure 8:**
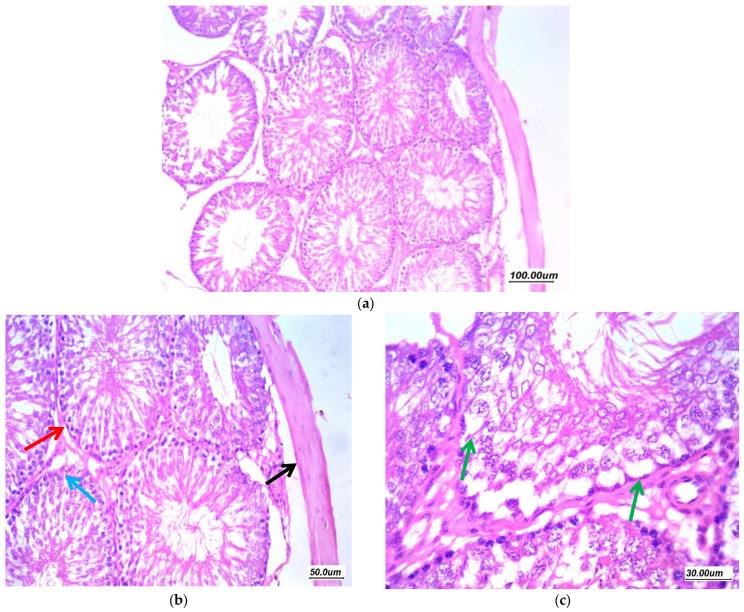
Effect of Pioglitazone (10 mg/kg/day) on gentamycin induced testicular damage in group (GIV, PIO10) (**a**) 100×, (**b**) 200×, and (**c**) 400×. There is a moderate improvement of changes, tubules showed regular arrangement with regular maturation (red arrow), moderate residual vacuolar degeneration of germ cells in few tubules (green arrow). Mild residual edema (black arrow) and thickening of tunica (black arrow).

**Figure 9:**
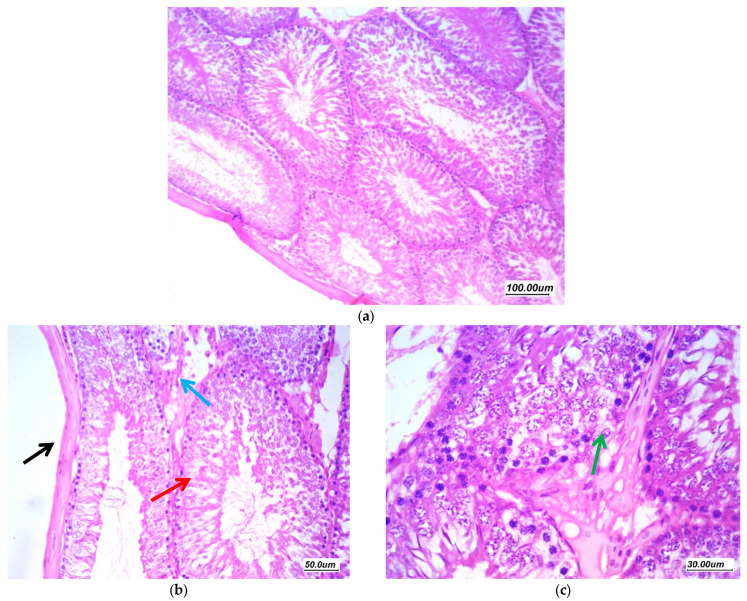
Effect of Pioglitazone (20 mg/kg/day) on gentamycin induced testicular damage in group (GV, PIO20) (**a**) 100×, (**b**) 200×, and (**c**) 400×. There is marked improvement of pathological changes, tubules are regularly arranged with regular contours, regular germ cell lining with regular maturation till spermatozoa stage (red arrow). There is focal minimal vacuolar degeneration of few cells (green arrow). There is mild focal interstitial edema (blue arrow) and mildly thickened tunica (black arrow).

Histopathological findings of the testicular tissue from different study groups are illustrated here (from Figure 5, Figure 6, Figure 7, Figure 8 and Figure 9). Figure 5 showed normal testicular tissue in the normal control group (GI, CTL). Sections revealed testicular tissue with regularly arranged seminiferous tubules with thin basement membrane lined by germ cells from basal spermatogonia showing regular maturation to the spermatid and spermatozoa stage. There are scattered Sertoli cells with associated spermatozoa. The interstitial tissue showed scattered Leydig cells and the Johnsen’s scoring system of testicular tissue was 10.

As presented in (Figure 6), H and E-stained sections of the gentamycin induced testicular damage group (GII, GM) showed markedly distorted seminiferous tubules showing variability in size and shape with irregular contours. There was marked distortion of germ cell lining with vacuolar degeneration, atrophy and shedding, loss of maturation and germ cells, with few tubules cystically dilated. There was moderate thickening of the basement membrane and moderate thickening of tunica. The interstitium showed marked edema as excess accumulation of extracellular fluid in the intertubular interstitial tissue with wide spacing between stroma cells and interstitial components, the Johnsen’s scoring system of testicular tissue was 3. 

Pioglitazone treated group (5 mg/kg/day) (GIII, PIO5) displayed regain of the normal histological appearance. There was a moderate improvement of changes, tubules showed regular arrangement with few showed irregular contours, moderate residual vacuolar degeneration of germ cells in few tubules. Moderate residual edema and focal thickening of the tunica as shown in (Figure 7), the Johnsen’s scoring system of testicular tissue was 8. 

Pioglitazone treated group (10 mg/kg/day) (GIV, PIO10) revealed nearly normal architecture as displayed in (Figure 8) There was a moderate improvement of changes, tubules showed regular arrangement with regular maturation, few tubules showed irregular contours, moderate residual vacuolar degeneration of germ cells in few tubules and mild residual edema, the Johnsen’s score was 8. 

Pioglitazone treated group (20 mg/kg/day) (GV, PIO20) revealed normal architecture. There was marked improvement of pathological changes, tubules are regularly arranged with regular contours, regular germ cell lining with regular maturation till spermatozoa stage. There was focal minimal vacuolar degeneration of a few cells. There was no focal interstitial edema as displayed in (Figure 9), the Johnsen’s scoring system of the testicular tissue was 9. 

Johnsen’s scoring system of testicular tissue in different groups was presented as scattered plot, as shown in Figure 12.

#### 3.7.2. Morphometric Assessment

**Figure 10:**
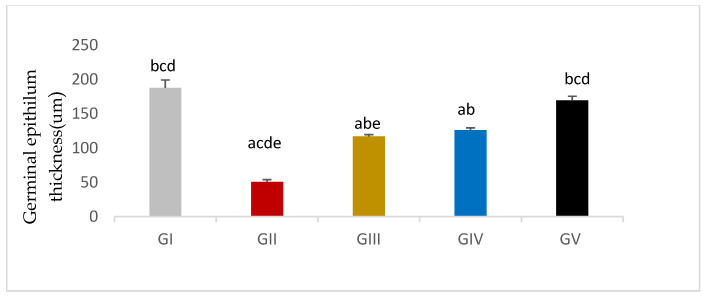
Germinal epithelium thickness in different groups, GI (CTL): normal control, GII (GM): gentamycin induced testicular damage, GIII (PIO5): pioglitazone treated (5 mg/kg/day), GIV (PIO10): pioglitazone treated (10 mg/kg/day) and GV (PIO20): pioglitazone treated (20 mg/kg/day). Values are as mean ± SEM, (^a^) compared to control group, (^b^) compared to diseased group, (^c^) compared to PIO 5 (GIII) group, (^d^) compared to PIO 10 (G IV) group (^e^) compared to PIO 20 (GV) group.

Germinal epithelium thickness was significantly reduced in response to gentamycin induction in (GII, GM) compared to GI, CTL (*p* < 0.01). While the treated groups (GIV, GV) showed a significant increase in germinal epithelium thickness compared to GII, GM (*p* < 0.01), group (GV, PIO20) showed a non -significant difference compared to (GI, CTL) (*p* > 0.05) (Figure 10, Table 12).

**Figure 11:**
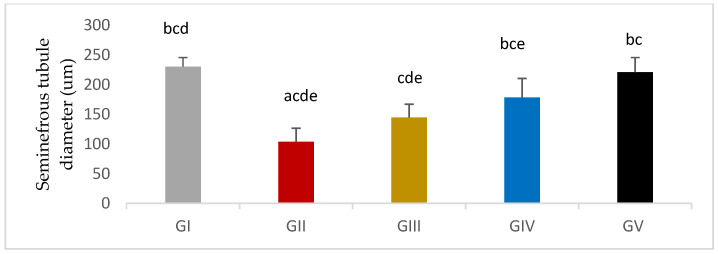
Seminiferous tubule thickness in different groups, GI (CTL): normal control, GII(GM): gentamycin induced testicular damage, GIII(PIO5): pioglitazone treated (5 mg/kg/day), GIV(PIO10): pioglitazone treated (10 mg/kg/day) and GV(PIO20): pioglitazone treated (20 mg/kg/day). Values are as mean ± SEM, (^a^) compared to control group, (^b^) compared to diseased group, (^c^) compared to PIO 5 (GIII) group, (^d^) compared to PIO 10 (G IV) group, (^e^) compared to PIO 20 (GV) group.

**Figure 12:**
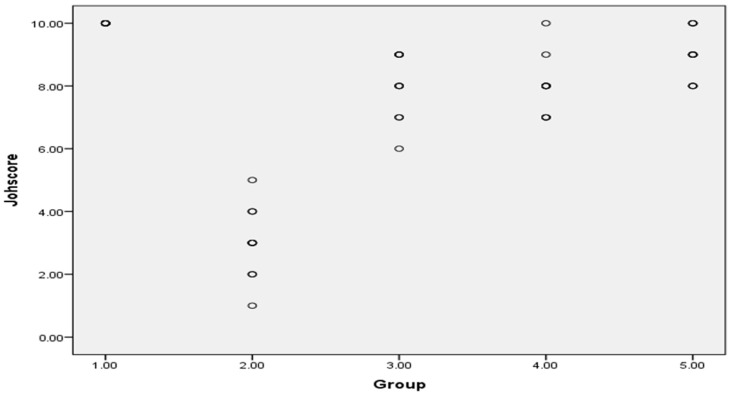
Johnsen’s scoring system of testicular tissue in different groups as scattered plot, G 1(CTL): normal control, G 2(GM): gentamycin induced testicular damage, G 3(PIO5): pioglitazone treated (5 mg/kg/day), G 4(PIO10): pioglitazone treated (10 mg/kg/day) and G 5(PIO20): pioglitazone treated (20 mg/kg/day). Values are as mean ± SEM.

Seminiferous tubule diameter was significantly decreased in response to gentamycin induction in (GII, GM) compared to GI, CTL (*p* < 0.01). While the treated groups (GIII, GIV, GV) showed a significant increase in seminiferous tubule diameter compared to GII, GM (*p* < 0.01) (Figure 11 and Figure 12).

## 4. Discussion

In previous studies, gentamycin was found to induce multi-organ damage such as hepatic toxicity, ototoxicity, and nephrotoxicity [20,35,36]. Moreover, PIO was previously approved to inhibit nephrotoxicity induced by gentamycin [20] Therefore, this study investigated whether PIO has a protective effect against testicular toxicity.

The present study elucidates the potential protective effect of PIO in testicular damage induced by gentamycin. The effect of gentamycin on the reproductive system of male Wistar albino rats was studied using the average dose of 100 mg/kg daily for six days [3]. Different doses of the PPAR-γ agonist, PIO, were used to investigate its protective effects against testicular damage induced by gentamycin.

Basically, gentamycin is related to aminoglycosides. It has a bactericidal action and causes an inhibition of bacterial protein synthesis by binding irreversibly to the 30 S bacterial ribosome. The first site of action is the outer bacterial membrane. The antibiotic particles make clefts in the outer bacterial cell membrane, leading to leakage of the bacterial cellular contents and increasing the uptake of gentamycin [37].

The current study showed that gentamycin-induced testicular damage in male albino Wistar rats caused a significant reduction in absolute testicular weight as well as relative testicular weight compared to the control group (*p* < 0.01 and *p* < 0.05). These findings are also confirmed with a study using doses of 60, 80, and 100 mg/kg for 10 days (*p* < 0.05), which reported a significant reduction in relative testicular weight with the higher two doses compared to control groups (*p* < 0.05) [6]. Several studies reported the same effect on testicular weight [38,39]. Another study used gentamycin in a dose of 100 mg/kg/day for six days and found no significant difference in testis and seminal vesicle weight (*p* > 0.05) [40].

The weight of the testes is mainly dependent on the mass of the differentiated spermatogenic cells and spermatozoa [41]. The decrease in the weight of the testes in this study may be due to a decrease in serum testosterone, inhibition of spermatogenesis, and as a result, decline in sperm production. The decreased serum testosterone in rats receiving gentamycin may be due to Leydig cell impairment caused by ROS generation and suppression of antioxidant activities, leading to inhibition of spermatogenesis and infertility at the end [39].

The decrease in the weight of testes due to germ cell loss matches our histopathological examination that revealed markedly distorted seminiferous tubules and germ cell lining with vacuolar degeneration, shedding, and loss of maturation. This was associated with a decrease in Johnsen’s score to 3. Hand in hand with our study, several studies found histopathological signs of testicular cell loss together with germ cell loss [6,38,42,43].

All of the adverse effects caused by gentamycin were improved in the subsequent histopathological sections following PIO treatment, and the improvement was dose-dependent reflected by testis weight since PIO treatment caused a significant increase in absolute testis weight compared to the gentamycin-treated group. Both treated groups GIV (PIO10) and GV (PIO 20) showed a significant increase in relative testis weight; GIII (PIO5) showed a non- significant difference. This is congruent with Jalilvand, N., et al., (2019), who found that the testis weight was elevated in PIO-treated hypothyroid rats following a dose of 20 mg/kg/day, compared to the hypothyroid group, but the difference was non-significant [44]. In the present study, there was significant improvement of Johnsen’s score matched with other reported improvement in the pathological picture and an increased Johnsen’s score of spermatogenesis with PIO treatment [45]. Oxidative stress, induced by gentamycin, is reversed by PIO supplementation through the inhibition of lipid peroxidation improving histopathological findings [46]. Also, the current work showed that elevated germinal epithelium thickness and decreased seminiferous tubule diameter caused by gentamycin could be prevented by PIO as reported in previous researches [47,48].

In the present study, the gentamycin group resulted in altered sperm parameters such as a significant decrease in the count, motility, and viability of the sperm compared to the control group (*p* < 0.01) with significant reduction of Johnsen’s score to 3. This negative effect of gentamycin on sperm parameters was also reported by others [3,6,38,40]. Reduction in sperm parameters due to gentamycin treatment could be attributed to a decreased level of serum testosterone, production of sperms in testis, and maturation of sperms in epididymis. Therefore, a reduction in sperm parameters is under the control of testosterone, due to increased testicular oxidative stress or both factors [3,49].

On the other hand, PIO-treated groups in different doses showed significant improvement in sperm parameters as was described by a study group that investigated PIO effects on testicular tissue damages in hypothyroidism induced by propylthiouracil in a rat model. It was found that PIO-treated hypothyroid rats showed a significant increase in sperm count compared to a hypothyroid group [44]. Also, it was found that PIO treatment in diabetic rats caused a significant rise in the count and motility of sperm, and a significant reduction in abnormal forms compared to a control diabetic rats’ group [47]. These improvements in sperm parameters and serum testosterone level were attributed to the antioxidant effects of PIO.

The original source of local androgen is Sertoli cells and Leydig cells which are the main controllers of spermatogenesis. Testosterone secretion is regulated by LH which binds to the androgen receptors of Sertoli cells and stimulates spermatogenesis [50]. Testosterone is crucial the production and maturation of normal sperm. A decrease in testosterone levels leads to failure of spermatogenesis and infertility [51]. Testosterone deficiency can also block spermatogenesis during miosis [52]. Rats with abnormal low levels of testosterone could not sustain Sertoli cell attachment to the spermatids and caused premature release and/or apoptosis of germ cells [53,54].

Regarding the pituitary-testicular hormonal axis, gentamycin has an inhibitory effect on steroidogenesis which leads to a significant drop in serum testosterone levels [55]. This was manifested by the reduction in the weight of testis, count, and the motility of sperm. Treatment with PIO significantly protected the testes from damage that could be induced by gentamycin. The testosterone level was significantly improved after PIO treatment until there was no significant variation between the control group and GV, PIO20. These findings suggested that PIO exhibited a protective effect on spermatogenesis. Reduction of the testosterone level may be related to gentamycin oxidative stress which affects different cell types and has a direct gonadotoxic effect [43,56].

In addition, the gentamycin-treated group showed a significant elevation in serum LH and FSH levels compared to the control group (*p* < 0.01). The serum LH and FSH levels decreased after PIO treatment. These results were in line with other previous studies which found that serum testosterone level was significantly decreased in gentamycin-treated groups [6,38,42,57]. The serum FSH and LH levels were both significantly elevated in all gentamycin-treated groups [38]. On the other hand, the serum FSH level was measured in an experimental study that examined the effects of gentamycin. This study recognized a significant decrease in serum testosterone, although the serum FSH level was not significantly affected [58]. The upsurge in LH and FSH may be due to decreased feedback inhibition of testosterone [59]. Consequently, modulation of peripheral testosterone, FSH, and LH might supply a treatment strategy for male infertility [60].

On studying the effect of gentamycin and PIO on inflammatory markers, this study found that the level of inflammatory markers IL-6 and TNF-α in the gentamycin-induced testicular damage group were significantly raised compared to the control group, while the level of these markers in the PIO-treated groups showed significant reduction (*p* ˂ 0.01) compared to the gentamycin-treated group. Similar findings were illustrated in a model of testicular damage induced by torsion/detorsion (T/D) that noticed an increase in TNF–α and IL-6 levels in the T/D group compared to sham. The levels of these inflammatory mediators in the PIO-treated group were reduced compared to the T/D group [45]. The protective effect of the PPAR-γ agonist on TNF–α and IL-6 was described in earlier studies about forebrain ischemia/reperfusion injury [61] and injury caused by myocardial ischemia/reperfusion [62]. PIO inhibits the inflammatory cytokines expression and makes the immune cells to be in anti-inflammatory phenotypes [18].

In germ cells, polyunsaturated fatty acids are required for energy production and cell membrane structure. These molecules are vulnerable to oxidative damage. Testis and epididymis germ cells are rich in these molecules, so these cells are enhanced with potent scavenger systems [63]. One possible mechanism for gentamycin-induced testicular cell damage is oxidative stress. ROS causes damage of sperm and other structures of the cytoplasmic organelle membrane as phospholipids, proteins, and nucleotides by peroxidation, leading to altering the motility of sperm [64]. In addition, ROS produces oxidative stress by reducing enzymatic defenses [65].

We found that induction of testicular damage in male rats by gentamycin caused a significant increase in the MDA level and a significant increase in TOS in the gentamycin-treated group. The previously mentioned effect was reported by other studies [3,39]. Pioglitazone treatment in this study significantly protected the testis against the damage caused by gentamycin. That result was in line with previous studies that established the inhibitory effect of PIO supplementation on lipid peroxidation [45,46,49,66]. Furthermore, GV (PIO20) showed a significant reduction in the MDA level compared to the control group. Further studies should be conducted to determine the beneficial effects of this therapeutic dose on other health conditions.

In this study, the gentamycin-treated group showed a significant reduction in tissue catalase and glutathione peroxidase activities which have a basic detoxification role against toxic metabolites. Also, a significant decrease in TAC with a significant increase in TOS and an increase in OSI were found. A study found that gentamycin treatment significantly reduced the activities of superoxide dismutase, catalase, glutathione peroxidase, glutathione reductase, glutathione-s-transferase, and the level of total reduced GSH compared to the control, which was similar to the results of the present study [3,39]. In the current study, treatment with PIO using PIO10 and PIO20 doses showed a significant increase in tissue catalase and glutathione peroxidase activities compared to the control, accompanied by a significant increase in TAC as well as a significant decrease in TOS. Also, OSI showed a significant reduction compared to the gentamycin-treated group (*p* < 0.01). It was mentioned that PIO treatment increased the activity of reduced glutathione, superoxide dismutase, and TAC with a concomitant decrease in TOS [45]. Contrarily, in the renal tissues of diabetic rats, it was found that there was no promising effect of PIO on the antioxidant enzyme activities such as catalase and reduced glutathione or the inflammatory markers such as TNF-α and IL-6 [67].

Pioglitazone has an antioxidant effect by decreasing ROS and elevating the antioxidant defense similarly to what was reported by a previous study which investigated PIO effects on testicular damage in a hypothyroid model and found that PIO-treated hypothyroid rats showed a significant reduction in MDA levels with elevation of the concentration of total thiol groups, SOD and CAT activities in testicular tissue compared to a hypothyroid-diseased group [44] The same effect was reported in diabetic rats testes [68]. This antioxidant effect of PIO was reported in other organs such as the liver [46].

One possible mechanism explaining the pathogenic effects of gentamycin on testicular cells is oxidative stress due to increased ROS production with a reduction in the activities of the antioxidant defense system [6]. ROS can impair cell membranes and important macromolecules such as lipids, proteins, and nucleic acid which eventually can lead to cell apoptosis. Nuclear DNA and mitochondria can be directly damaged by ROS. They can activate P53 or C-Jun N-terminal Kinase (JNK), which can interfere with the activity of anti-apoptotic proteins [10,69].

Apoptosis is a physiological procedure that occurs normally during spermatogenesis; however, a rise in the ROS level enhances the intrinsic apoptotic pathway through elevation of the pro-apoptotic Bax gene expression and declining anti-apoptotic Bcl-2 with consequent mitochondrial membrane damage [70]. Consequently, mitochondrial cytochrome-C is released into the cytoplasm and stimulates a cascade of caspases comprising caspase-3 which triggers caspase-activated DNase to destroy DNA [71]. Furthermore, TNF-α can trigger an extrinsic pathway of apoptosis [72]. The sum of these events leads to apoptosis in the germ cells.

In the existing study, the expression of the BAX apoptotic gene was significantly increased in the gentamycin-treated group and decreased with PIO treatment. On the other hand, expression of the anti-apoptotic gene BCL2 showed a significant elevation in the PIO-treated the groups. It has been noticed that PIO amplified Bcl-2 expression in the testicular tissue of T/D testicles that can be proposed as an anti-apoptotic effect of PIO and also decreased caspase 3 activity [73] It was also found that in a type 2 model of diabetic rats, PIO treatment significantly increased the Bcl-2 area in immunohistochemical staining and significantly reduced caspase-3 gene expression, suggesting anti-apoptotic activity [73]. Furthermore, the reno-protective influence of PIO against ischemia/re-perfusion injury was reported and attributed to augmentation of Bcl-2 expression, which antagonizes the apoptotic activity of Bax. This results in preservation of mitochondrial action and cell membrane integrity [74].

## 5. Conclusions

In conclusion, pioglitazone of 20 mg/kg/day could protect testicular damage in germinal and non-germinal cells from the toxic effects of gentamycin through its antioxidant, anti-inflammatory, and anti-apoptotic effects. PIO inhibited testicular damage through the preservation of histopathological architecture, and increased the endogenous testosterone levels and maintenance of overall spermatozoal parameters. To the best of our knowledge, this study is the first that gives an insight into the mode of action and potential protective effect of PIO on testicular damage. It could open up new prospects for many people who anticipate using gentamycin without worrying about its testicular side effects.

## Figures and Tables

**Table 1:** Scheme of the experimental groups.

**Group**	PIO	GM
Group I (CTL)	“–”	“–”
Group II	–	100 mg/kg/day for 6 days
Group III	5 mg/kg/day for 21 days	100 mg/kg/day for 6 days started on day 15 of PIO treatment
Group IV	10 mg/kg/day for 21 days	100 mg/kg/day for 6 days started on day 15 of PIO treatment
Group V	20 mg/kg/day for 21 days	100 mg/kg/day for 6 days started on day 15 of PIO treatment

**Table 2:** The set of primers of polymerase chain reaction (PCR) for the selected genes.

Primer	Sequence	Annealing Temperature
BAX	FOR: 5′-CCAGTTGAAGTTGCCGTCTG-3′ REV: 5′-AAGAAGCTGAGCGAGTGTCT-3′	56
BCL2	FOR: 5′- ATGTGTGTGGAGAGCGTCAA-3′ REV: 5′-GATGCCGGTTCAGGTACTCA-3′	57
GAPDH	FOR: 5′-CTCTCTGCTCCTCCCTGTTC-3′ REV: 5′-TACGGCCAAATCCGTTCACA-3′	60

**Table 3:** Johnsen’s scoring system a histopathological examination of testicular tissue.

Scheme.	Score Description
1	No cell
2	Sertoli cells without germ cells
3	Only spermatogonia
4	Only a few spermatocytes
5	Many spermatocytes
6	Only a few early spermatids
7	Many early spermatids without differentiation
8	Few late spermatids
9	Many late spermatids
10	Full spermatogenesis

**Table 4:** Effects of gentamycin induced testicular damage and pioglitazone treatment on body weight, testis weight and relative testis weight.

Group Variable (Mean ± SEM)	GI (*n* = 10)	GII (*n* = 10)	GIII (*n* = 10)	GIV (*n* = 10)	GV (*n* = 10)
Body weight (gm)	232.80 ± 8.84	229.50 ± 6.89	241.60 ± 8.47	230.50 ± 5.57	236.40 ± 4.39
Absolute testis weight (mg)	131.50 ± 0.50 ^b^	106 ± 0.53 ^acde^	124.50 ± 2.72 ^be^	133.30 ± 3.47 ^b^	141.9 ± 1.96 ^bc^
Relative testis weight	0.58 ± 0.04 ^b^	0.47 ±0.03 ^ade^	0.52 ± 0.02	0.58 ±0.01 ^b^	0.60 ±0.01 ^b^

GI: CTL, normal control, GII: GM, gentamycin induced testicular damage, GIII: PIO 5 (5 mg/kg/day) pioglitazone treated group, GIV: PIO 10 pioglitazone treated (10 mg/kg/day) and GV: PIO 20 pioglitazone treated (20 mg/kg/day). Values are expressed as mean ± SEM. (^a^) compared to control group (GI), (^b^) compared to GM treated group (GII), (^c^) compared to PIO 5 (GIII) group, (^d^) compared to PIO 10 (G IV) group, (^e^) compared to PIO 20 (GV) group.

**Table 5:** Effects of gentamycin induced testicular damage and pioglitazone treatment on sperm parameters.

Group	GI (*n* = 10)	GII (*n* = 10)	GIII (*n* = 10)	GIV (*n* = 10)	GV (*n* = 10)
Sperm count (×10^6^)	62.23 ± 0.88 ^bcd^	4.97 ± 0.37 ^acde^	54.28 ± 0.44 ^be^	55.85 ± 0.52 ^abe^	60.18 ± 0.56 ^bcd^
Sperm motility (%)	84.22 ± 0.78	65.37 ± 0.91 ^a^	72.89 ± 0.91 ^b^	78.57 ± 0.43 ^b^	82.85 ± 0.89 ^b^
Sperm viability (%)	80.6 ± 1.71	59.69 ± 0.32 ^a^	65.58 ± 0.58 ^b^	71.6 ± 0.96 ^b^	76.20 ± 1.67 ^b^

GI: CTL, normal control, GII: GM, gentamycin induced testicular damage, GIII: PIO 5 pioglitazone treated (5 mg/kg/day), GIV: PIO 10 pioglitazone treated (10 mg/kg/day) and GV: PIO 20 pioglitazone treated (20 mg/kg/day). Values are expressed as mean ± SEM, (^a^) compared to control group (GI), (^b^) compared to GM treated group (GII), (^c^) compared to PIO 5 (GIII) group, (^d^) compared to PIO 10 (G IV) group, (^e^) compared to PIO 20 (GV) group.

**Table 6:** Effects of gentamycin induced testicular damage and pioglitazone treatment on serum testosterone, LH and FSH levels.

Group Variable	GI (*n* = 10)	GII (*n* = 10)	GIII (*n* = 10)	GIV (*n* = 10)	GV (*n* = 10)
Serum Testosterone(ng/mL)	1.74 ± 0.06 ^cd^	0.61 ± 0.02 ^acde^	1.01 ± 0.14 ^abe^	1.15 ± 0.05 ^abc^	1.62 ± 0.03 ^bcd^
Serum LH (mIU/mL)	0.56 ± 0.02 ^bc^	1.69 ± 0.05 ^acde^	1.30 ± 0.05 ^abde^	0.68 ± 0.05 ^bc^	0.62 ± 0.02 ^bc^
Serum FSH (mIU/mL)	1.25 ± 0.03 ^bcd^	3.32 ± 0.09 ^acde^	2.49 ± 0.08 ^abde^	1.64 ± 0.06 ^abce^	1.3 ± 0.04 ^bcd^

GI: CTL, normal control, GII: GM, gentamycin induced testicular damage, GIII: PIO 5 pioglitazone treated (5 mg/kg/day), GIV: PIO 10 pioglitazone treated (10 mg/kg/day) and GV: PIO 20 pioglitazone treated (20 mg/kg/day). Values are expressed as mean ± SEM (^a^) compared to control group, (^b^) compared to a diseased group, (^c^) compared to PIO 5 (GIII) group, (^d^) compared to PIO 10 (G IV) group, (^e^) compared to PIO 20 (GV) group.

**Table 7:** Effects of gentamycin induced testicular damage and pioglitazone treatment on serum TNF-α and IL-6 levels.

Group Variable	GI (*n* = 10)	GII (*n* = 10)	GIII (*n* = 10)	GIV (*n* = 10)	GV (*n* = 10)
TNF-α (pg/mL)	11.80 ± 0.43 ^bcde^	79.66 ± 0.89 ^acde^	36.80 ± 0.49 ^abde^	32.23 ± 0.39 ^abde^	20.93 ± 1.03 ^abc^^d^
IL-6 (pg/mL)	13.68 ± 0.52 ^bcde^	87.20 ± 1.53 ^acde^	66.05 ± 0.52 ^bde^	46.75 ± 3.41 ^b^^de^	32.18 ± 0.29 ^b^^cd^

GI: CTL, normal control, GII: GM, gentamycin induced testicular damage, GIII: PIO 5 pioglitazone treated (5 mg/kg/day), GIV: PIO 10 pioglitazone treated (10 mg/kg/day) and GV: PIO 20 pioglitazone treated (20 mg/kg/day). Values are expressed as mean ± SEM, (^a^) compared to control group (GI), (^b^) compared to GM treated group (GII), (^c^) compared to PIO 5 (GIII) group, (^d^) compared to PIO 10 (G IV) group, (^e^) compared to PIO 20 (GV) group.

**Table 8:** Effects of gentamycin induced testicular damage and pioglitazone treatment on total antioxidant capacity (TAC), total oxidative stress (TOS) and oxidative stress index (OSI) serum levels.

Group Variable	GI (*n* = 10)	GII (*n* = 10)	GIII (*n* = 10)	GIV (*n* = 10)	GV (*n* = 10)
TAC (mM/L)	1.53 ± 0.10 ^bc^	0.91 ± 0.05 ^ade^	1.13 ± 0.03 ^abe^	1.3 ± 0.04 ^b^	1.48 ± 0.11 ^b^^c^
TOS (mM/L)	0.32 ± 0.03 ^bcd^	2.19 ± 0.10 ^acde^	1.32 ± 0.05 ^abde^	1.07 ± 0.02 ^abe^	0.33 ± 0.03 ^bcd^
OSI	0.22 ± 0.03 ^bcd^	2.47 ± 0.15 ^acde^	1.17 ± 0.05 ^abde^	0.84 ± 0.03 ^abce^	0.25 ± 0.04 ^bcd^

GI: CTL, normal control, GII: GM, gentamycin induced testicular damage, GIII: PIO 5 pioglitazone treated (5 mg/kg/day), GIV: PIO 10 pioglitazone treated (10 mg/kg/day) and GV: PIO 20 pioglitazone treated (20 mg/kg/day). Values are expressed as mean ± SEM, (^a^) compared to control group (GI), (^b^) compared to GM treated group (GII), (^c^) compared to PIO 5 (GIII) group, (^d^) compared to PIO 10 (G IV) group, (^e^) compared to PIO 20 (GV) group.

**Table 9:** Effects of gentamycin induced testicular damage and pioglitazone treatment on malondialdehyde (MDA) levels.

Group Variable (Mean ± SEM)	GI (*n* = 10)	GII (*n* = 10)	GIII (*n* = 10)	GIV (*n* = 10)	GV (*n* = 10)
MDA (nmol/g)	34.66±0.99 ^bce^	49.08±2.55 ^acde^	43.05±0.95 ^abde^	35.42±1.02 ^bce^	29.42±0.54 ^abcd^

GI: CTL, normal control, GII: GM, gentamycin induced testicular damage, GIII: PIO 5 pioglitazone treated (5 mg/kg/day), GIV: PIO 10 pioglitazone treated (10 mg/kg/day) and GV: PIO 20 pioglitazone treated (20 mg/kg/day). Values are expressed as mean ± SEM, (^a^) compared to control group (GI), (^b^) compared to GM treated group (GII), (^c^) compared to PIO 5 (GIII) group, (^d^) compared to PIO 10 (G IV) group, (^e^) compared to PIO 20 (GV) group.

**Table 10:** Effects of gentamycin induced testicular damage and pioglitazone treatment on tissue activities of glutathione peroxidase and catalase in different groups.

Group Variable (Mean ± SEM)	GI (*n* = 10)	GII (*n* = 10)	GIII (*n* = 10)	GIV (*n* = 10)	GV (*n* = 10)
CAT (u/gm)	3.24 ± 0.11 ^bcde^	2.17 ± 0.03 ^ade^	2.21 ± 0.02 ^ae^	2.95 ± 0.05 ^abce^	2.67 ± 0.04 ^abcd^
GP (u/mg)	179.83 ± 1.39 ^bcd^	161.92 ± 0.88 ^ade^	163.49 ± 1.27 ^ade^	172.46 ± 2.29 ^abce^	179.49 ± 1.65 ^bcd^

GI: CTL, normal control, GII: GM, gentamycin induced testicular damage, GIII: PIO 5 pioglitazone treated (5 mg/kg/day), GIV: PIO 10 pioglitazone treated (10 mg/kg/day) and GV: PIO 20 pioglitazone treated (20 mg/kg/day). Values are expressed as mean ± SEM, (^a^) compared to control group (GI), (^b^) compared to GM treated group (GII), (^c^) compared to PIO 5 (GIII) group, (^d^) compared to PIO 10 (G IV) group, (^e^) compared to PIO 20 (GV) group.

**Table 11:** Effects of gentamycin induced testicular damage and pioglitazone treatment on mRNA Expression levels of BAX and BCL2 genes in different groups.

Group Variable (Mean ± SEM)	GI (*n* = 10)	GII (*n* = 10)	GIII (*n* = 10)	GIV (*n* = 10)	GV (*n* = 10)
BAX	1.01 ± 0.01 ^bcde^	6.26 ± 0.21 ^acd^	3.43 ± 0.14 ^abde^	2.73 ± 0.05 ^abce^	2.05 ± 0.03 ^abcd^
BCL2	0.99 ± 0.08 ^cde^	0.79 ± 0.07 ^cde^	2.22 ± 0.09 ^abde^	2.86 ± 0.08 ^abce^	4.17 ± 0.25 ^abcd^

GI: CTL, normal control, GII: GM, gentamycin induced testicular damage, GIII: PIO 5 pioglitazone treated (5 mg/kg/day), GIV: PIO 10 pioglitazone treated (10 mg/kg/day) and GV: PIO 20 pioglitazone treated (20 mg/kg/day). Values are expressed as mean ± SEM, (^a^) compared to control group (GI), (^b^) compared to GM treated group (GII), (^c^) compared to PIO 5 (GIII) group, (^d^)compared to PIO 10 (G IV) group, (^e^) compared to PIO 20 (GV) group.

**Table 12:** Effects of gentamycin induced testicular damage and pioglitazone treatment on Germinal epithelium thickness and Seminiferous tubule diameter in different groups.

Group Variable (Mean ± SEM)	GI (*n* = 10)	GII (*n* = 10)	GIII (*n* = 10)	GIV (*n* = 10)	GV (*n* = 10)
Germinal epithelial thickness	187.93 ± 11.35 ^bcd^	50.73±3.32 ^acde^	117.15±2.49 ^abe^	126.31±3.29 ^ab^	169.86±5.98 ^bcd^
SNT diameter	230.32 ± 4.84 ^bcd^	103.89 ± 7.18 ^acde^	144.75±7.05 ^cde^	178.65±10.06 ^bce^	221.11±7.75 ^bc^

GI: CTL, normal control, GII: GM, gentamycin induced testicular damage, GIII: PIO 5 pioglitazone treated (5 mg/kg/day), GIV: PIO 10 pioglitazone treated (10 mg/kg/day) and GV: PIO 20 pioglitazone treated (20 mg/kg/day). Values are expressed as mean ± SEM, (^a^) compared to control group (GI), (^b^) compared to GM treated group (GII), (^c^) compared to PIO 5 (GIII) group, (^d^) compared to PIO 10 (G IV) group, (^e^) compared to PIO 20 (GV) group.

## Data Availability

Data is contained within the article.
